# Determining the Composition of Resident and Transient Members of the Oyster Microbiome

**DOI:** 10.3389/fmicb.2021.828692

**Published:** 2022-02-02

**Authors:** Andrea Unzueta-Martínez, Heather Welch, Jennifer L. Bowen

**Affiliations:** Department of Marine and Environmental Sciences, Northeastern University, Nahant, MA, United States

**Keywords:** animal microbiome, oyster microbiome, microbial community assembly, resident microbes, transient microbes, microbial ecology

## Abstract

To better understand how complex microbial communities become assembled on eukaryotic hosts, it is essential to disentangle the balance between stochastic and deterministic processes that drive their assembly. Deterministic processes can create consistent patterns of microbiome membership that result in persistent resident communities, while stochastic processes can result in random fluctuation of microbiome members that are transient with regard to their association to the host. We sampled oyster reefs from six different populations across the east coast of the United States. At each site we collected gill tissues for microbial community analysis and additionally collected and shipped live oysters to Northeastern University where they were held in a common garden experiment. We then examined the microbiome shifts in gill tissues weekly for 6 weeks using 16S rRNA gene amplicon sequencing. We found a strong population-specific signal in the microbial community composition of field-sampled oysters. Surprisingly, the oysters sampled during the common garden experiment maintained compositionally distinct gill-associated microbial communities that reflected their wild population of origin, even after rearing them in a common garden for several weeks. This indicates that oyster gill-associated microbiota are predominantly composed of resident microbes specific to host population, rather than being a reflection of their immediate biotic and abiotic surroundings. However, certain bacterial taxa tended to appear more frequently on individuals from different populations than on individuals from the same population, indicating that there is a small portion of the gill microbiome that is transient and is readily exchanged with the environmental pool of microbes. Regardless, the majority of gill-associated microbes were resident members that were specific to each oyster population, suggesting that there are strong deterministic factors that govern a large portion of the gill microbiome. A small portion of the microbial communities, however, was transient and moved among oyster populations, indicating that stochastic assembly also contributes to the oyster gill microbiome. Our results are relevant to the oyster aquaculture industry and oyster conservation efforts because resident members of the oyster microbiome may represent microbes that are important to oyster health and some of these key members vary depending on oyster population.

## Introduction

Host-associated microbial communities play vital roles in host development, nutrition, and even behavior (McFall-Ngai et al., 2013). Because of their ubiquity and importance to host physiology, the field of biology has been challenged to re-think what constitutes an individual organism (Rohwer et al., 2002; Rosenberg et al., 2007; Gordon et al., 2013; Bordenstein and Theis, 2015) and how animals evolve (Rosenberg and Zilber-Rosenberg, 2013). The undeniable importance of animal microbiomes has led human medical researchers to focus on the human microbiome (Kashyap et al., 2017) and wildlife conservation biologists to include host-associated microbiome knowledge into wildlife management practices (Trevelline et al., 2019). Host-associated microbiomes are governed by a variety of dynamic microbe-microbe, host-microbe, and environment-microbe interactions (Konopka, 2009; Bauer et al., 2018). However, to gain a better understanding of the ecology and evolution of animals and their microbiomes, it is also essential to investigate the ecological drivers that structure their microbial communities.

Microbial community assembly can be influenced by stochastic processes involving random birth, death, and immigration events (Hubble, 2001), as well as deterministic processes involving selection by biotic and abiotic factors (Chesson, 2000). Stochastic processes (e.g., neutral community assembly) can explain microbial community dynamics in some free-living microbial assemblages, where random immigration plays an important role in shaping microbial communities (Ofiteru et al., 2010). Alternatively, microbial community structure can be shaped by deterministic factors like environmental heterogeneity, which is evident in soil microbial populations, where the variable environment allows for extensive niche partitioning and diverse communities (Ramette and Tiedje, 2007). Most studies on microbial community assembly mechanisms suggest that both deterministic and stochastic processes play significant roles in structuring microbial communities.

The assembly of animal-associated microbial communities is shaped by multiple interacting factors including host genetics, age, and environment (Benson et al., 2010; Yatsunenko et al., 2012). Many host-associated microbial communities, for example those found in wasps (Brucker and Bordenstein, 2012), termites (Dietrich et al., 2014), and mammals (Sanders et al., 2014), can parallel the phylogeny of the host species (Brooks et al., 2016), suggesting strong host factors are at play. There is also evidence that environmental factors, such as diet, can play a significant role in host microbial community composition (David et al., 2014; McFrederick et al., 2017). Changes in diet can result in a portion of the microbiome that varies among individuals and reflects host dietary choice (McFrederick et al., 2017). The degree to which interacting factors contribute to shaping host-associated microbial communities remains unclear for many different host-microbe systems.

To gain a better understanding of the forces that structure animal microbiomes, we must determine which members of the microbiome are strictly governed by the host, and which members are governed by external environmental conditions or by stochastic processes that are unrelated to the host. Microbial members that are explicitly selected for by the host can be considered resident associates of their host, whereas microbial members driven by external factors (e.g., the local environment or stochastic processes) can be considered transient associates of their host. For example, some oyster hemolymph bacteria such as *Vibrio* spp. can persist, despite the high filtration activity of oysters, in the absence of an environmental source population (e.g., when held in sterile seawater) and over a range of environmental conditions, suggesting that host factors maintain this resident relationship (Vasconcelos and Lee, 1972; Lokmer et al., 2016a,b). On the opposite side of the spectrum, the green macroalgae *Ulva australis* has a surface microbiome that is highly variable between individuals and lacks a core microbiome (Burke et al., 2011), suggesting that more neutral forces govern this microbiome assemblage, which results in a transient association with their host. There are also examples of animal microbiomes that fall between these extremes, shrimp larvae share a portion of their microbiome with their surrounding seawater bacterioplankton but are ultimately distinct from it (Wang et al., 2020), suggesting that shrimp larvae microbiomes may contain members that are resident associates of their shrimp host and others that are more transient and change with changes to the seawater environment.

We used the Eastern oyster (*Crassostrea virginica*) as a model to study host-associated microbial community assembly dynamics in a marine bivalve. Eastern oysters are an important fisheries species with high commercial value (Fisheries and Agriculture Organisation of the United Nations [FAO], 2018) and are critically important to the structure and function of estuarine habitats (Burge et al., 2014), as a result there has been substantial research devoted to their biology, physiology, ecology, immunology, and more recently their associated microbial community ecology. Oysters have diverse microbiomes that are tissue type specific (King et al., 2012; Li et al., 2017), and that vary depending on geographic location (Lokmer et al., 2016a). Additionally, wild oysters can be easily sampled from their habitat and can be successfully reared in the laboratory, making them ideal for coupled field and laboratory experiments.

To differentiate between resident and transient members of the oyster microbiome, we collected oysters from six wild populations, sacrificing some instantaneously to assess oyster-associated microbiomes at each site. We housed the remaining representatives from those populations in a common environment, and monitored shifts in their gill microbiomes through 6 weeks in the common garden. To determine whether there were resident members of the oyster gill microbiome, we assessed whether (1) the field oysters harbored microbial communities that were different depending on population, and if (2) those differences were maintained throughout the 6 week common garden. To determine whether there were transient members of the oyster gill microbiome, we assessed whether (3) there was within population divergence of the common garden oyster microbes from their field representatives. To disentangle which members of the oyster microbiome where resident and which where transient, we adapted a metric designed to assess lineage fidelity (Moeller et al., 2018) and applied it to disentangle resident vs. transient members of the microbiome to assess whether (4) all oyster microbiome members displayed the same degree of host association. Parsing out resident and transient members of the oyster microbiome will provide insight into the balance between stochastic and deterministic forces driving community dynamics in this commercially important marine species.

## Materials and Methods

### Field Oyster Collections

At low tide, we collected adult *C. virginica* (80–100 mm shell length) from six different intertidal oyster reefs along the East Coast of the United States in the summer of 2018. In the northeastern Atlantic Bight, we sampled at the Damariscotta River (44°01′38.1″N 69°32′35.7″W) in Maine (ME), Barnstable (41°42′37.6″N 70°18′18.5″W) in Massachusetts (MA), and Green Hill Pond (41°22′16.1″N 71°37′13.4″W) in Rhode Island (RI). In the southeastern Atlantic Bight we sampled from Horse Island (37°17′15.5″N 75°55′02.0″W) in Virginia (VA), Atlantic Beach (34°42′24.9″N 76°45′05.7″W) in North Carolina (NC), and St. Augustine (29°40′17.7″N 81°12′53.5″W) in Florida (FL). We selected these sites based on previous work suggesting significant genetic differentiation among populations across these geographical regions (Hoover and Gaffney, 2005; Hughes et al., 2017). At each site, we shucked and dissected five oysters to collect their gill tissues for microbial community analysis. We chose to sample gill tissues because they are constantly in contact with the surrounding seawater microbiome through filtering and as a result, are likely to have consistent exposure to local microbial sources. We sterilized our dissecting tools with ethanol and, once collected, tissue samples were thoroughly rinsed with autoclave-sterile water, flash frozen in liquid nitrogen, and stored at −80°C until DNA extraction. An additional 32 oysters were collected at each site and transported on ice to Northeastern University to conduct the common garden experiment.

### Common Garden

We conducted a common garden experiment at Northeastern University’s Marine Science Center, using a flow-through seawater system that draws water from Broad Sound in Nahant, Massachusetts. We housed oysters from each population independently of the other populations, in 21 L tanks. We chose to keep populations in separate tanks because we were interested in seeing whether oysters would take up microbes from their environment without adding more noise to this signal by mixing populations together. All populations were exposed to the same filtered seawater at the same rate (150 mL per minute, which replaced all the seawater in each tank every 2 h), and they were maintained at 17°C and aerated with air stones for the duration of the experiment. The oysters were fed 20 mL of a 1% Shellfish Diet 1800^®^ solution twice daily, following best practices outlined in Helm and Bourne (2004). Each population was replicated across four independent tanks, with eight oysters per tank, and we sampled each population at 24, 48 h, and once weekly for 6 weeks upon arrival. We chose this resolution because we wanted to capture short- and long-term changes in the oyster microbiomes. At each timepoint, we shucked and dissected four oysters per population (each from an independent tank) to collect their gill tissues for microbial community analysis. All of the collected tissue samples were thoroughly rinsed with autoclave-sterile water, flash frozen in liquid nitrogen, and stored at −80°C until DNA extraction.

### Nucleic Acid Preparation and Sequencing

We extracted DNA from the oyster gills using the DNeasy PowerLyzer PowerSoil kit (Qiagen, Valencia, CA United States) following the manufacturer’s procedure. To characterize the microbiomes associated with gill tissues, we amplified the V4 region of the 16S rRNA gene using, the primers 515FY: 5′TATGGTAATTGTGTGYCAGCMGCCGCGGTAA3′ (Parada et al., 2016) and 806RB: 3′ AGTCAGTCAGCCGGACT ACNVGGGTWTCTAAT 5′ (Apprill et al., 2015) in triplicate 25 μL PCR reactions. These primers were chosen based on their fairly comprehensive coverage of prokaryotes. Samples were amplified with the following thermocycler conditions: a 3 min hot start at 94°C followed by 35 cycles of 94°C for 45 s, 50°C for 60 s, and 72°C for 90 s. The final extension step was 72°C for 10 min. We then checked the triplicate PCR product and negative controls on a gel to ensure there was no contamination and that the PCR product matched the target size of ∼390 bp. We purified and size selected the PCR product using Agencourt AMPure Magnetic Beads (Beckman Coulter, Brea, CA, United States), and resuspended them in 20 μL of nuclease-free water. We used Nextera XT v2 indexes to ligate Illumina paired-end adapters to 2 μL of 16S rRNA amplicons using 8 cycles of PCR. The thermocycler conditions were as follows: a 3 min hot start at 95°C followed by 8 cycles of 95°C for 30 s, 55°C for 30 s, 72°C for 30 s, and a final extension step at 72°C for 5 min. We then purified and size selected the PCR products using Agencourt AMPure Magnetic Beads, and resuspended them in 20 μL of nuclease-free water. We used Quant-iT PicoGreen dsDNA Assay Kit (Invitrogen, Carlsbad, CA, United States) to quantify our libraries and pooled them at equimolar concentrations. We confirmed library size on an Agilent 4200 TapeStation (Agilent Technologies, Santa Clara, CA, United States), quantified the library using a KAPA library quantification kit (Roche Sequencing Solutions Inc., Pleasanton, CA, United States) and sequenced our libraries on an Illumina MiSeq with 2×250 V2 sequencing chemistry at the Tufts University Core Sequencing Facility.

### Procedural Controls

We collected and sequenced procedural controls to ensure the quality of our data set. During the library preparation process we included and sequenced DNA extraction negative controls and PCR amplification negative controls with every batch. Additionally we sequenced four replicates of a mock community (ZymoBIOMICS™ Microbial Community DNA Standard, Zymo Research, United States), with known theoretical relative abundances of 10 species, as positive control. Our mock community replicates were highly consistent with their expected composition (Supplementary Figure 1).

### Sequencing Analysis

For each sequencing run, we used the DADA2 (v1.7.0) workflow with default parameters (Callahan et al., 2016), implemented in R Studio (v4.0.0), to quality-filter, merge paired-end reads, remove chimeric sequences, and group the sequences into amplicon sequence variants (ASVs). We then merged the ASV tables from both sequencing runs and assigned taxonomy against the Silva database (version 132; Quast et al., 2012). We used the Phyloseq package (McMurdie and Holmes, 2013) for initial processing of the ASV table and identified potential procedural and reagent contaminants using the decontam package based on either the frequency of each ASV as a function of the input DNA concentration or the prevalence of each ASV in true samples compared to the prevalence in negative controls (Davis et al., 2018). Additionally we filtered out ASVs that were identified as chloroplasts, Eukaryota, and Archaea, which accounted for less than 0.1% of our data set. Samples with less than 1,000 sequences were excluded.

### Statistical Analysis

To test our first two hypotheses of whether (1) field oysters harbored microbial communities that were different depending on oyster population, and if (2) those differences were maintained throughout the 6 week common garden, we focused on β-diversity and computed a Sorensen-Dice dissimilarity matrix using the vegdist function in Vegan (Oksanen et al., 2020). For the field and common garden samples, we independently ran a multivariate analysis of variance (PERMANOVA) with 999 permutations using adonis2 and tested for homogeneity of group dispersions using the betadisper function in Vegan (Oksanen et al., 2020).

We compared common garden samples to field samples, both across the entire common garden experiment and specifically at the last time point (6 weeks), and compared the mean Sorensen-Dice dissimilarities within and between populations using a two-samples Wilcoxon test (Gehan, 1965), with the wilcox.test function in base R. Additionally, we visualized the Sorensen-Dice dissimilarities within and between populations of common garden samples with violin plots.

To test our third hypothesis that there was within population divergence of the common garden oyster microbes from their field representatives, we compared Sorensen-Dice dissimilarities between common garden samples to field samples within the same oyster population over the course of the experiment. We plotted these comparisons over time and computed a linear regression model to assess whether there was a significant turnover of the microbial community with time. We hypothesized that if a specific population had a more transient microbiome, they would become more dissimilar from their initial population with time. We performed this analysis for all common garden samples together and for each population independently to examine population specific differences.

To test our fourth hypothesis that all oyster microbiome members displayed the same degree of association to their hosts, we modified a metric used to determine the degree of fidelity of bacterial ASVs to specific host lineages (Moeller et al., 2018) and applied it to examine the degree of flexibility of the oyster microbiome (ranging from strictly-associated resident microbes to loosely associated transient microbes). We used both field and common garden samples and only considered ASVs that were present in more than 1% of the samples (*n* = 1,375 ASVs). We calculated the mean Sorensen-Dice dissimilarity for each bacterial ASV between individuals from different populations and between individuals from the same population. We defined a flexibility score (FS) as the ratio of within-population to between-population Sorensen-Dice dissimilarities. A FS > 1 indicates that the ASV tended to be restricted to specific populations (resident association with oyster population), FS < 1 indicated that the ASV was more often shared by individuals from different populations than individuals from the same population (transient association with the oyster host), and FS = 1 indicated that the ASV was equally distributed among individuals regardless of population (Moeller et al., 2018). We then used a density plot and Hartigan’s dip test, to assess whether the distribution of flexibility scores was unimodal. We visualized the distribution and abundance patterns of ASVs with high and low FS in three different ways. First we looked at the FS distribution of ASVs at the order level using box and whisker plots, then we visualized the average relative abundance of ASVs with the top 1% and bottom 1% FS (*n* = 28) using stacked bar plots, and lastly we visualized relative abundance patters over time of ASVs with the top 1% FS (*n* = 14) with connected scatter plots. All figures were created in ggplot2 (Wickham, 2016).

## Results

**FIGURE 1:**
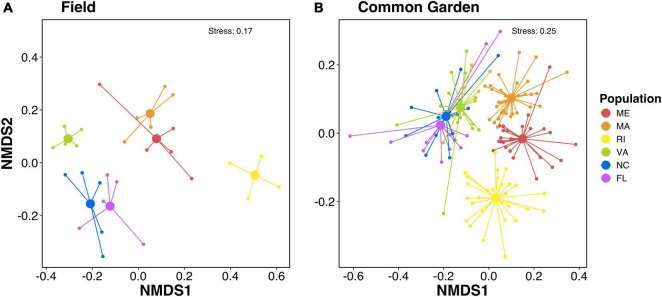
Non-metric multidimensional scaling (NMDS) plots of sorensen-dice dissimilarities of microbial communities associated with the gills of **(A)** field and **(B)** common garden oysters. PERMANOVA results show significant effects of population on microbial community composition for both field (*p* < 0.001) and common garden (*p* < 0.002). Colors represent oyster population.

We found that oyster gills harbored compositionally distinct microbial communities depending on their population. Non-metric multidimensional scaling (NMDS) ordination plots of the Sorensen-Dice dissimilarity indices showed microbial communities clustering by population in field samples (Figure 1A) and in common garden samples (Figure 1B). Permutational multivariate analysis of variance (PERMANOVA) showed significant effects of population on microbial community composition for both field (*p* < 0.001; Supplementary Table 1) and common garden (*p* < 0.002; Supplementary Table 2) samples. Additionally, there is separation between the northern (ME, MA, RI) and southern (VA, NC, FL) populations along the NMDS1 axis in both the field and common garden samples (Figures 1A,B).

**FIGURE 2:**
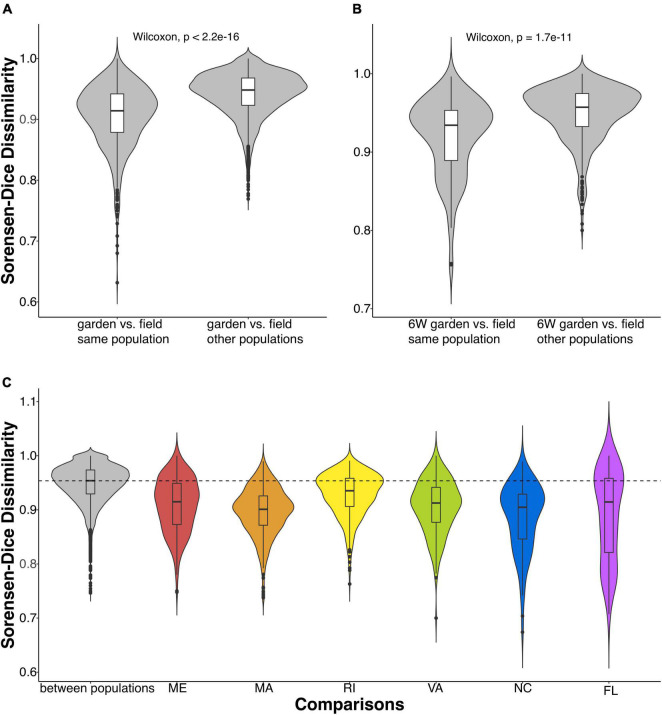
Violin plots comparing Sorensen-Dice dissimilarities of gill-associated microbial communities between and within oyster populations. **(A)** Comparisons of common garden vs. field oysters within and between populations (Wilcoxon, *p* < 2.2e-16). **(B)** Comparisons of oysters at the last time point (in the common garden for 6 weeks) and oysters collected in the field between and within populations (Wilcoxon, *p* < 1.7e-11). **(C)** Common garden samples compared between populations (in gray) and within each population. The dashed line indicates the mean of the between-population Sorensen-Dice dissimilarity.

We then compared the Sorensen-Dice dissimilarities of common garden samples to field samples. The microbiomes of common garden samples tended to be more similar to those of individuals sampled in their own population of origin than to individuals from a different population (Figure 2). A Wilcoxon test showed that the mean dissimilarity of within-population comparisons were more similar to each other than the mean dissimilarities of between-population comparisons (*p* < 2.2e-16; Figure 2A). Even when we compared only the final time point, 6 weeks after sharing a common environment, common garden oysters were still more similar to their field counterparts than to other populations (*p* < 1.7e-11; Figure 2B). When we examined the population specific response, we observed a similar pattern where the mean dissimilarities of within-population comparisons were always more similar than between-population comparisons (Figure 2C).

**FIGURE 3:**
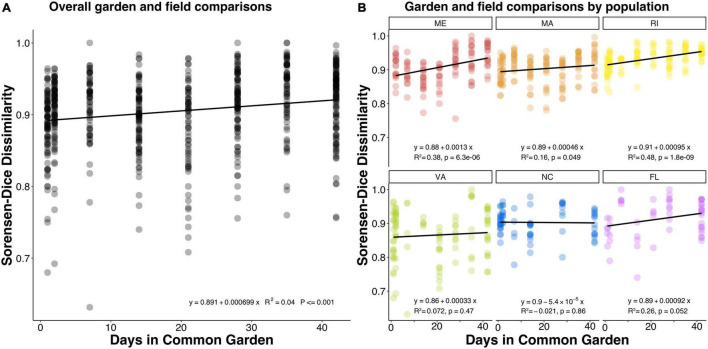
Dot plots showing Sorensen-Dice dissimilarities comparing common garden samples to field samples within the same oyster population over the course of the experiment. Each dot represents a comparison between one common garden sample and one field sample both belonging to the same population. **(A)** The overall pattern for all populations, **(B)** the patterns of each population separately. A positive slope indicates that common garden samples became more dissimilar from their field population representatives over time.

We compared common garden samples to their field representatives within the same population at each timepoint and found that despite their within-population similarity, over time the microbial communities of common garden oysters did become increasingly different from their field representatives (Figure 3A; *p* = 0.001). Despite the extent of variation, the significantly positive slope indicates that the dissimilarities were slightly increasing over time across all populations. We also examined these patterns within each population and observed a mixed response, with half of the populations becoming more dissimilar from their field representatives (Figure 3B). Interestingly, the common garden samples from ME, MA, and RI, significantly changed over time from their field representatives, whereas the common garden samples from VA, NC, and FL did not significantly change.

**FIGURE 4:**
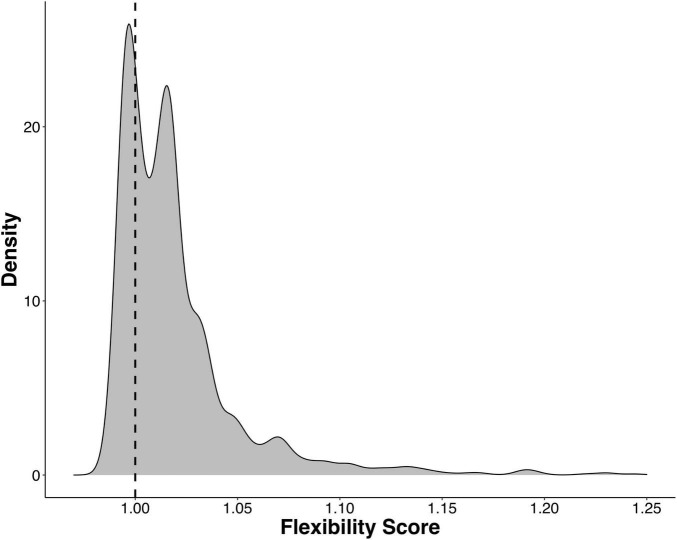
Kernel density plot that shows the distribution of flexibility scores for all ASVs that were detected in more than 1% of the samples (*n* = 1375). Hartigan’s dip test for unimodality, *p* < 2.2e-16, indicates a bimodal distribution. Dashed line denotes a flexibility score of 1.

**FIGURE 5:**
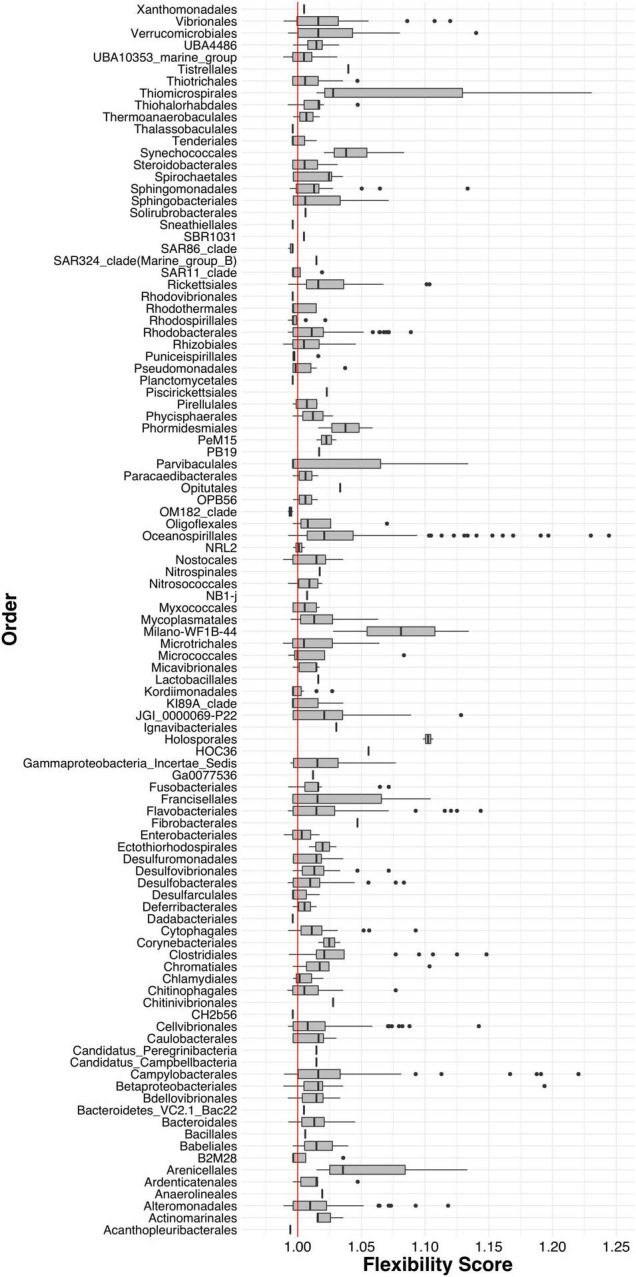
Box and whiskers plots of the flexibility scores of ASVs grouped by Order. Red line indicates a flexibility score of 1.

Next, we calculated a flexibility score (FS) to determine which ASVs were transient and which were resident to their host after being reared in a common environment. We found that the majority of gill-associated ASVs displayed a flexibility score > 1, indicating that they were resident. However, the distribution of flexibility scores was bimodal (Hartigan’s dip test *p* < 2.2e-16) with a dip around 1.01 (Figure 4), indicating a number of ASVs that were either shared by two or more populations or were evenly distributed across all populations. When visualizing the distribution of flexibility scores for ASVs at the order level, we found that some orders, such as Thiomicrospirales, Holosporales, and Arenicellales had all ASVs with a flexibility score > 1 (Figure 5). In contrast, some orders, such as Ancathopleuribacterales, CH2b6, and Dadabacteriales, had all ASVs with a flexibility score < 1 (Figure 5). We also found that within the same order, ASVs could display mixed flexibility scores (Figure 5).

**FIGURE 6:**
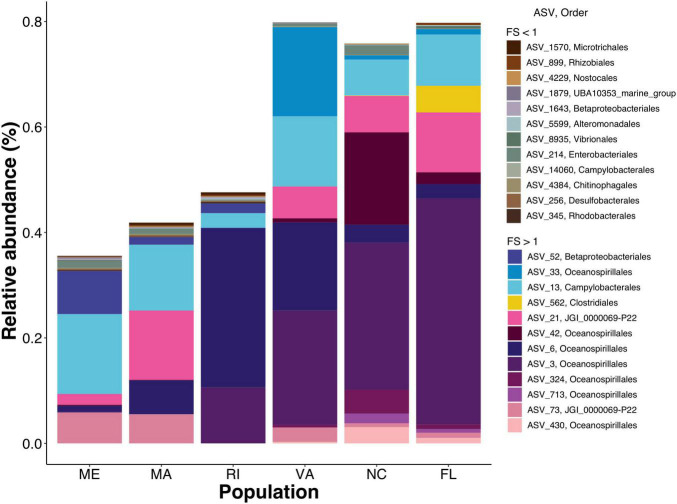
Staked bar plot of the mean relative abundances by population of ASVs with the top and bottom 1% FS. Earth tones represent ASVs with FS < 1 vibrant colors represent FS > 1. Detailed taxonomic information for these ASVs available in Supplementary Table 3.

**FIGURE 7:**
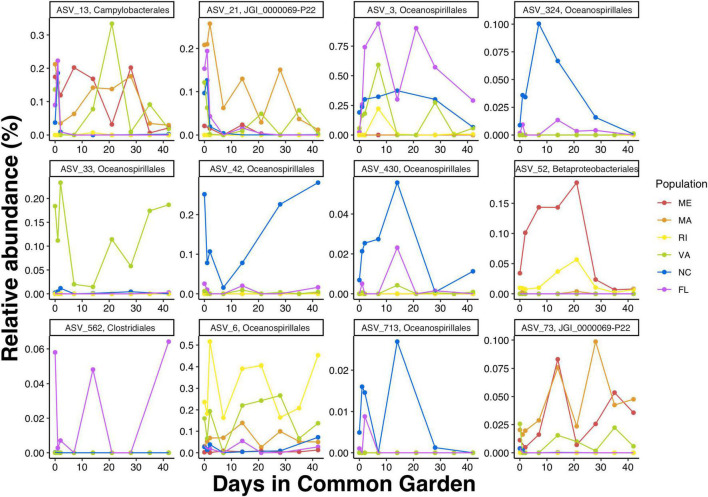
Mean relative abundances by timepoint of ASVs with the top 1% FS (i.e., FS > 1). Detailed taxonomic information form these ASVs is available in Supplementary Table 3.

To get a closer look at individual ASVs, we plotted ASVs with FS in the top and bottom 1% of the distribution (*n* = 28). Out of these 28 ASVs, we found that ASVs with FS > 1 were much more abundant than ASVs with FS < 1, and that some ASVs were unique to a specific population while others were shared among multiple populations (Figure 6 and Supplementary Table 3). Consistent with the regression results that showed northern populations having greater change over time than southern populations, the cumulative relative abundance of the resident taxa was considerably higher in the southern populations. Next we plotted ASVs with the highest FS (top 1%) across time to examine the temporal shifts in the resident ASVs. We found abundance patterns that persisted within specific populations over time but were often not shared among populations. For example, ASV_33, in the order Oceanospirillales, family *Nitricolaceae*, was present in the field and at all timepoints in the common garden oysters from VA, but was not present in the majority of oysters from the five other populations (Figure 7).

## Discussion

The diverse collections of microorganisms that inhabit animal hosts are critical to host health, but the mechanisms underlying their assembly are poorly understood. To work toward gaining a better understanding of the potential microbial community assembly mechanisms on oyster gills we aimed to differentiate between resident and transient members of the oyster microbiome. We found that (1) field-collected oysters harbored microbial communities that were different depending on population, and surprisingly (2) those differences were maintained throughout the 6 week common garden. This suggests that a considerable portion of the complex oyster microbiome is resident according to oyster population. Additionally, we found that (3) there was a small degree of within population divergence of the common garden oyster microbes from their field representatives in some populations, suggesting that there is small proportion of the microbiome that is transient and readily exchanged with their environmental pool of microbes.

Since gill microbiomes displayed evidence of both resident and transient members, we proceeded to disentangle the degree of flexibility of specific bacterial ASVs (ranging from strictly-associated resident microbes to loosely associated transient microbes). To disentangle which members of the oyster microbiome were resident and which were transient, we calculated a flexibility score (FS) and found that (4) the majority of oyster-gill ASVs were resident to their oyster population, rather than being a reflection of the bacterioplankton in their immediate surroundings. However, a few low abundance bacterial taxa tended to appear more frequently on individuals from different populations than on individuals from the same population over time, indicating that a small proportion of the complex gill microbiome is transient and readily exchanged with the environment. These results provide insight into the relative contributions of deterministic and stochastic processes to the microbial community assembly of adult-oyster gills.

### β Diversity Patterns Indicate Resident Members of the Oyster Microbiome

We found that gill microbiomes of oysters collected in the field were significantly different depending on oyster population. This finding is consistent with previous studies that found a strong relationship between geographic location and oyster microbiota (King et al., 2012; Nguyen et al., 2020). We selected our sites based on previous work indicating significant genetic differentiation among populations across these geographical regions (Hoover and Gaffney, 2005; Hughes et al., 2017). It is possible that the microbiome differences we observed may be due to the genetic differences among populations, as host genetics play important roles in microbiome composition (Turnbaugh et al., 2009; McKnite et al., 2012; Bonder et al., 2016; Goodrich et al., 2016). Alternatively, microbiomes of field oysters may have differed by population due to the different biotic and abiotic factors at the geographic location where each population was collected. Previous studies found that environmental factors like temperature (Lokmer and Mathias Wegner, 2015) and pH (Scanes et al., 2021) can influence oyster-associated microbial communities. However, our results indicating population fidelity through the common garden experiment support the critical role of genetic populations in determining oyster microbiome composition.

We found that gill-microbiome differences by population persisted throughout the 6 week common garden experiment. After 6 weeks of being exposed to the same biotic and abiotic environmental conditions, oyster gill microbiomes were still more similar to those within the same population than between populations. This finding is consistent with previous literature that found that invasive oysters can retain gill microbiomes that are more similar to those of oysters from their range of origin than to the microbiomes of co-occurring oysters (Zurel et al., 2011; Roterman et al., 2015). We can conclude from these results that host factors were more important than environmental factors in shaping oyster-gill microbiomes in our experiment.

### β Diversity Patterns Indicate Transient Members of the Oyster Microbiome

Even though host factors were dominant in shaping oyster-gill microbiomes, we also found β diversity patterns that indicated that there was a small degree of exchange between oyster-gill microbes and the seawater bacterioplankton in the common garden. We found that overall, the gill microbiomes of common garden oysters became slightly more dissimilar from their field counterparts within the same population over time, although the variance in this relationship was remarkably high. The slight increase in dissimilarity can be attributed to oysters acquiring microbes from the common garden seawater microbial community, which is consistent with oyster reciprocal transplant studies that found transient bacteria that were derived from the seawater bacterioplankton (Lokmer et al., 2016a,b). Environmental bacteria that can integrate into an animal’s microbiome are commonly reported in microbiome studies including those of sponges (Blanquer et al., 2013), and mammalian guts (Zhang et al., 2016). However, our data suggest that the uptake of environmental microbes might be population-specific.

Surprisingly, we found that northern populations from ME, MA, and RI significantly changed over time from their field representatives, whereas the common garden oyster samples from VA, NC, and FL did not significantly change (Figure 3). This finding was particularly surprising because our common garden was conducted in the state of MA (north Atlantic Bight), where we might expect experimental conditions to be more similar to field conditions of northern populations than those of southern populations. Northern and southern populations were differentiated along the primary axis in NMDS dissimilarity plots, both in the field collected samples (Figure 1A) and after the common garden (Figure 1B), suggesting fundamental and persistent differences between the southern and northern populations. Southern populations show a greater proportion of taxa that are resident to their host (Figure 6), consistent with the lack of change over time. It is possible that this pattern results from greater similarities among the common garden conditions and the biotic and abiotic conditions found at the northern sites, which might then facilitate greater exchange between the host and the microbes available in the common garden experiment. Alternatively, northern populations withstand stronger seasonal variations in temperature, which can exert control over oyster microbiomes (Lokmer and Mathias Wegner, 2015). Thus, northern populations could be primed to shift their microbiomes with changing environmental conditions. Further investigation is required to determine the nature of microbiome exchange with familiar and unfamiliar environmental pools of microbes and whether that could explain the geographical split we observed. Repeating the common garden experiment in one of the southern locations, thereby giving the southern populations a more familiar environment, would provide a rigorous test of this hypothesis. If familiarity with the microbiome facilitates greater turnover, we would expect the patterns to be reversed and see a smaller amount of microbiome turnover in the northers populations.

### Flexibility of Specific Bacterial Amplicon Sequence Variants

Since gill microbiomes displayed evidence of both resident and transient members, we proceeded to disentangle the degree of flexibility of specific bacterial ASVs. We found that the majority of ASVs in our experiment were resident to their oyster population, i.e., they were more often found among oysters within the same population than across multiple populations, even at the end of the common garden experiment. This could potentially be explained by host factors, like genetics, that can form consistent patterns in oyster gill microbiome composition depending on oyster genotype (King et al., 2012, 2019; Nguyen et al., 2020). Our findings are also consistent with studies that have found that oyster microbiomes are composed of mostly resident microbes and not as many transient microbes that are exchanged with seawater bacterioplankton (Lokmer et al., 2016a,b). In our study, the distribution of flexibility scores (FS) across all ASVs was bimodal with a dip around one (Figure 4), suggesting that bacterial ASVs on oyster gills were either resident or transient with relatively few ASVs displaying mixed flexibility modes.

When looking at distribution of FS for ASVs grouped at the order level, we found substantial within-order variability. Most orders had ASVs with mixed FS, indicating that broader taxonomic classifications can mask ASV-level patterns of resident or transient association to their host oyster and highlighting the importance of fine taxonomic resolution to resolve differences among microbiomes of marine invertebrates, as previously described (Reveillaud et al., 2014). We also found that the most strictly-associated resident ASVs (top 1% of the distribution) had a considerably higher abundance than transient ASVs. ASVs with low abundances are not necessarily transient, however, as low abundance taxa can play important roles in shaping microbial communities (Berry and Widder, 2014; Herren and McMahon, 2018) and can be persistent at low abundance among individuals in a population (Adair et al., 2018; Antwis et al., 2019). We also detected specific ASVs that were highly persistent within individual populations or among multiple populations. Calculating FS helped us identify specific resident ASVs and this method provides a unique mechanism for differentiating host-selected microbes, from those that reflect their immediate environment.

Some of the ASVs identified in the top 1% of the FS distribution (ASV_230, ASV_713, ASV_6, and ASV_42) were members of the order Oceanospirillales, family *Endozoicomonadaceae*, genus *Endozoicomonas*. Members of this genus are known for their symbiotic associations with a wide variety of marine organisms including invertebrates such as cnidarians, porifera, and mollusks (Fiore et al., 2015; Morrow et al., 2015; Roterman et al., 2015). Marine invertebrate-associated *Endozoicomonas* genomes suggest that they can contribute to the cycling of carbohydrates and provision of proteins to their animal host (Neave et al., 2017). Members of this genus can also produce bioactive secondary metabolites that could play a role in regulating bacterial colonization of their animal host (Jessen et al., 2013; Rua et al., 2014; Morrow et al., 2015; reviewed in Neave et al., 2016). Additionally, *Endozoicomonas* ASVs have been identified as dominant in oysters resistant to QX-disease (Nguyen et al., 2020). The nutritional, signaling, and protective properties of this bacterial genus could be fundamental to host health and thus make good resident members of the oyster microbiome. Another ASV identified in the top 1% of the FS distribution (ASV_52) belongs to the order Betaprotobacteriales, family *Burkholdericeae.* Members of this family have been found in symbiotic associations with insects (Takeshita and Kikuchi, 2017) and have also been found to persist on different oyster species, across multiple life stages, and geographical regions in the Gulf of Mexico (Trabal et al., 2012). Host-associated *Burkholdericeae* strains are known to promote growth and protect their plant host from disease (Bevivino et al., 1998). It is possible the oyster-associated *Burkholdericeae* play similar roles and are therefore desirable resident members of the oyster microbiome. The functional importance of ASVs with high flexibility scores should be investigated due to their prevalence among oyster populations and their potential importance in promoting oyster fitness. Identifying microbes that potentially improve host fitness are critical to promoting sustainable oyster aquaculture and for the conservation and management of oyster populations.

## Conclusion

To disentangle the relative contributions of deterministic and stochastic processes to the microbial community assembly of adult oysters, we identified resident members and transient members of the oyster microbiome. We defined resident, as microbes that where highly persistent on oyster populations and were tightly associated to their oyster host. On the other hand, transient microbes were readily exchanged with the environmental pool of microbes and therefore were loosely associated with the oyster host. We found that the majority of adult oyster-gill microbes were resident to a specific host population, suggesting that deterministic processes such as host factors contribute substantially to the assembly of adult oyster-gill microbiomes. The smaller proportion of the community that was transient, suggests that environmental and stochastic processes that are beyond host control, contributed a smaller amount to the assembly of oyster-gill microbiomes. Additionally, we demonstrate the application of Flexibility Scores to disentangle, at the ASV level, which members of the microbiome are resident to their host population and which members are transient. Overall, our study shows that not all members of the oyster-gill microbiome are governed by the same ecological dynamics and it contributes to the efforts that seek to disentangle the mechanisms by which host-associated microbial communities become assembled. Lastly, our results also have implications for oyster conservation efforts as they demonstrate microbiome differences between oyster populations, which suggests conservations efforts may be more effective at a population level rather than species level.

## Data Availability Statement

The datasets presented in this study can be found in online repositories. The names of the repository/repositories and accession number(s) can be found below: https://www.ncbi.nlm.nih.gov/bioproject/783632?log, accession number PRJNA783632.

## Author Contributions

AU-M under the guidance of JB designed the experiments, collected and processed samples, conducted statistical analyses, and led the writing of the manuscript. HW conducted the experiments, collected, and processed samples. All authors contributed to the article and approved the submitted version.

## Conflict of Interest

The authors declare that the research was conducted in the absence of any commercial or financial relationships that could be construed as a potential conflict of interest.

## Publisher’s Note

All claims expressed in this article are solely those of the authors and do not necessarily represent those of their affiliated organizations, or those of the publisher, the editors and the reviewers. Any product that may be evaluated in this article, or claim that may be made by its manufacturer, is not guaranteed or endorsed by the publisher.
