# Supplementary material for: Determining the Composition of Resident and Transient Members of the Oyster Microbiome
Source: Front Microbiol. 2022 Feb 2;12:828692. doi: 10.3389/fmicb.2021.828692 (PMC8847785; doi:10.3389/fmicb.2021.828692)
Supplement: Supplementary file 1 [file Data_Sheet_1.PDF]

Determining the composition of resident and transient members of the oyster microbiome

Supplemental Information

Table S1. Statistical analysis of the effects of population on Sorensen-Dice dissimilarities of field oysters-associated microbial communities. A: Results of permutational test for homogeneity of group dispersions (betadisper), the only assumption of Adonis. B: Results of PERMANOVA (Adonis), using 999 permutations.

A. Homogeneity of group dispersionst test:

Factor	Df	Sum of Squares	Mean Square	F	Pr(>F)	significance
Populations	5	0.031084	0.0062167	2.305	0.07615	NS
Residuals	24	0.06473	0.0026971			

B. PERMANOVA:

Factor	Df	Sum of Squares	Mean Squares	F.Model	R2	Pr(>F)	significance
Populations	5	4.022	0.8044	2.6361	0.3545	0.001	***
Residuals	24	7.3236	0.30515	0.6455			
Total	29	11.3456	1				

Table S2. Statistical analysis of the effects of population on Sorensen-Dice dissimilarities of common garden oysters-associated microbial communities. A: Results of permutational test for homogeneity of group dispersions (betadisper), the only assumption of Adonis. B: Pairwise comparisons of the group dispersion test. C: Results of PERMANOVA (Adonis), using 999 permutations.

A. Homogeneity of group dispersions test:

Factor	Df	Sum of Squa	Mean Square	F	Pr(>F)	significance
Population	5	0.024859	0.0049718	2.8768	0.01707	*
Residuals	126	0.217758	0.0017282			

B. PERMANOVA:

Factor	Df	Sums of Squ	MeanSqs	F.Model	R2	Pr(>F)	significance
Pupulation	5	6.255	1.25104	3.0406	0.10767	0.001	***
Residuals	126	51.842	0.41145	0.89233			
Total	131	58.098	1				

Table S3. Taxonomic information, identified using the Silva database, of ASVs in the top (A) and bottom (B) 1% of the FS distribution.

A. ASVs with FS score in the top 1% of the distribution (FS>1)

ASV	Flexibility Score	Kingdom	Phylum	Class	Order	Family	Genus
ASV_430	1.346199815	Bacteria	Proteobacteria	Gammaproteobacteria	Oceanospirillales	Endozoicomonadaceae	Endozoicomonas
ASV_73	1.404447795	Bacteria	Patescibacteria	Gracilibacteria	JGI_0000069-P22	NA	NA
ASV_713	1.423640959	Bacteria	Proteobacteria	Gammaproteobacteria	Oceanospirillales	Endozoicomonadaceae	Endozoicomonas
ASV_324	1.46680191	Bacteria	Proteobacteria	Gammaproteobacteria	Oceanospirillales	Endozoicomonadaceae	NA
ASV_3	1.4762406	Bacteria	Proteobacteria	Gammaproteobacteria	Oceanospirillales	Nitrincolaceae	NA
ASV_6	1.563325743	Bacteria	Proteobacteria	Gammaproteobacteria	Oceanospirillales	Endozoicomonadaceae	Endozoicomonas
ASV_42	1.587214886	Bacteria	Proteobacteria	Gammaproteobacteria	Oceanospirillales	Endozoicomonadaceae	Endozoicomonas
ASV_21	1.670181022	Bacteria	Patescibacteria	Gracilibacteria	JGI_0000069-P22	NA	NA
ASV_562	1.776176276	Bacteria	Firmicutes	Clostridia	Clostridiales	Clostridiaceae_1	Clostridium_sensu_stricto_1
ASV_13	1.779151943	Bacteria	Epsilonbacteraeota	Campylobacteria	Campylobacterales	NA	NA
ASV_33	2.429850351	Bacteria	Proteobacteria	Gammaproteobacteria	Oceanospirillales	Endozoicomonadaceae	Endozoicomonas
ASV_52	2.704226217	Bacteria	Proteobacteria	Gammaproteobacteria	Betaproteobacteriales	Burkholderiaceae	NA

B. ASVs with FS score in the bottom 1% of the distribution (FS<1)

ASV	Flexibility Score	Kingdom	Phylum	Class	Order	Family	Genus
ASV_1570	0.988372093	Bacteria	Actinobacteria	Acidimicrobiia	Microtrichales	Ilumatobacteraceae	Ilumatobacter
ASV_899	0.988478878	Bacteria	Proteobacteria	Alphaproteobacteria	Rhizobiales	Devosiaceae	NA
ASV_4229	0.988505747	Bacteria	Cyanobacteria	Oxyphotobacteria	Nostocales	Oscillatoriaceae	Planktothricoides_SR001
ASV_1879	0.988657845	Bacteria	Proteobacteria	Gammaproteobacteria	UBA10353_marine_group	NA	NA
ASV_1643	0.98878553	Bacteria	Proteobacteria	Gammaproteobacteria	Betaproteobacteriales	Nitrosomonadaceae	Nitrosomonas
ASV_5599	0.988826816	Bacteria	Proteobacteria	Gammaproteobacteria	Alteromonadales	Alteromonadaceae	Paraglaciecola
ASV_8935	0.989032511	Bacteria	Proteobacteria	Gammaproteobacteria	Vibrionales	Vibrionaceae	Aliivibrio
ASV_214	0.989136802	Bacteria	Proteobacteria	Gammaproteobacteria	Enterobacteriales	Enterobacteriaceae	NA
ASV_14060	0.989249498	Bacteria	Epsilonbacteraeota	Campylobacteria	Campylobacterales	NA	NA
ASV_4384	0.99190091	Bacteria	Bacteroidetes	Bacteroidia	Chitinophagales	Saprospiraceae	NA
ASV_256	0.992167102	Bacteria	Proteobacteria	Deltaproteobacteria	Desulfobacterales	Desulfobacteraceae	Sva0081_sediment_group
ASV_345	0.992167102	Bacteria	Proteobacteria	Alphaproteobacteria	Rhodobacterales	Rhodobacteraceae	NA

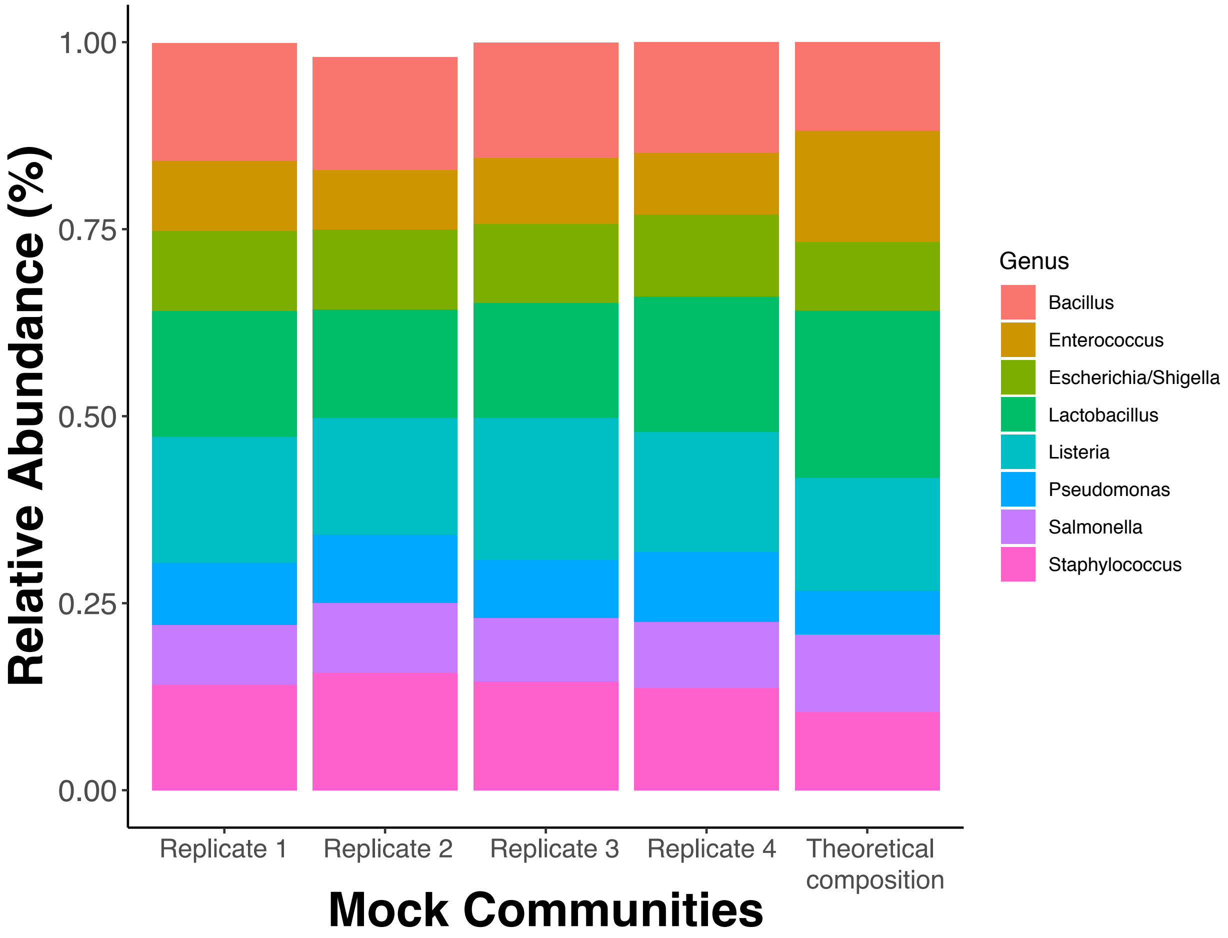

Figure S1. Stacked bar plot of the relative abundance of bacterial genera comprising mock community replicates and the theoretical composition. Relative abundance was calculated within each sample and ASVs that made up less than 1% of the sample were excluded.
